# Treatment of Relapsed/Refractory Hodgkin Lymphoma: Real-World Data from the Czech Republic and Slovakia

**DOI:** 10.7150/jca.29308

**Published:** 2019-08-28

**Authors:** Zdeněk Král, Jozef Michalka, Heidi Móciková, Jana Marková, Alice Sýkorová, David Belada, Alexandra Jungová, Samuel Vokurka, Marie Lukášová, Vít Procházka, Juraj Ďuraš, Roman Hájek, Ladislav Dušek, Ľuboš Drgoňa, Miriam Ladická, Veronika Ballová, Andrej Vranovský

**Affiliations:** 1 Department of Internal Medicine, Hematology and Oncology, University Hospital Brno and Faculty of Medicine, Masaryk University, Brno, Czech Republic; 2 Department of Clinical Hematology, University Hospital Kralovske Vinohrady and Third Faculty of Medicine, Charles University, Prague, Czech Republic; 3 4th Department of Internal Medicine - Hematology, University Hospital Hradec Kralove, Czech Republic and Charles University in Prague, Faculty of Medicine in Hradec Kralove, Czech Republic; 4 Department of Haemato-Oncology, University Hospital in Plzen, Plzen 304 60, Czech Republic; 5 Department of Hemato-Oncology, Faculty of Medicine and Dentistry, Palacký University, Olomouc, Czech Republic; 6 Department of Hemato-Oncology, Faculty of Medicine, University of Ostrava and University Hospital Ostrava, Ostrava, Czech Republic; 7 Institute of Biostatistics and Analyses, Faculty of Medicine, Masaryk University, Czech Republic; 8 Department of Oncohematology, Comenius University in Bratislava and National Cancer Institute, Bratislava, Slovakia; 9 Department of Hematology/Oncology, Kantonsspital Baden, Baden, Switzerland

**Keywords:** antibody-drug conjugate, CD30, brentuximab vedotin, Hodgkin lymphoma, registries, stem cell transplantation

## Abstract

**Introduction**: Clinical trials have demonstrated the effectiveness of the CD30-targeted antibody-drug conjugate brentuximab vedotin (BV) for the treatment of relapsed/refractory Hodgkin lymphoma (R/R HL). In this study, we report on outcomes with BV in a real-world setting using data collected in clinics in the Czech Republic and Slovakia.

**Patients and Methods**: Clinical and epidemiological data for patients with R/R HL who received treatment with BV at eight centers across the Czech Republic and Slovakia were examined. Data were amalgamated and analyzed retrospectively.

**Results**: Clinical data for 58 patients (median age: 30.5 years) with R/R HL who received BV during the course of their treatment were collected and analyzed. Patients had received a median of 3 prior treatment regimens and most (91%) were treated with BV after relapse following autologous stem cell transplantation. Therapeutic responses after BV included 19 (33%) complete responses (CRs) and 8 (14%) partial responses. CRs occurred more frequently in patients who had received fewer prior treatment regimens. The 1-, 2-, and 3-year overall survival (OS) rates from initiation of BV were 78%, 62%, and 41%, respectively.

**Conclusion**: Response rates and OS in this analysis of BV in real-world settings in the Czech Republic and Slovakia were consistent with those reported for pivotal clinical trials and from previous studies outside the clinical trial setting. The results support the efficacy of BV for treatment of R/R HL in real-life clinical practice.

## Introduction

Treatment for Hodgkin lymphoma (HL) achieves very high cure rates, with most patients (>80%) achieving a cure and long-term survival. However, approximately 20-40% of patients experience a relapse after front-line therapy or fail to respond to initial treatment, with approximately 50% of these patients being subsequently salvaged by high-dose chemotherapy followed by autologous stem cell transplantation (ASCT), which is the standard of care for most patients according to the European Society for Medical Oncology (ESMO) guidelines for the management of HL.^1^-^5^ In patients with failure after ASCT the outlook is poor, with a median survival of only 25 months.^6^

A number of factors that are predictive of outcome after ASCT have been identified, such as early (<12 months) relapse after ASCT, disease refractory to front-line therapy, failure to achieve a response to the most recent salvage therapy, extranodal disease (stage IV) or B-symptoms at pre-ASCT relapse, prior use of two or more salvage therapies, bulky disease, poor performance status, and age ≥50 years at relapse. Patients with one or more of these factors have worse outcomes after ASCT.^7^ Furthermore, some patients are not eligible for ASCT due to factors such as age, refractory disease, or poor performance status. For these patients, new treatment strategies are needed urgently.

Brentuximab vedotin (BV) is a CD30-targeting antibody-drug conjugate that was shown in a pivotal phase II trial to be an effective and well-tolerated treatment for patients with relapsed/refractory (R/R) HL after ASCT with an overall objective response rate (ORR) of 75% and complete remission in 34% of patients.^8^ Recently published 5-year follow-up data from the trial showed that durable responses could be achieved even without further anticancer therapy, with 9 of the 34 patients (26%) who achieved a complete response (CR) still in remission and potentially cured.^9^ Studies have also shown that BV is an effective option for patients with R/R HL who are ineligible for transplantation.^10^,^11^

While clinical trials are critical for establishing efficacy, collection of real-world data outside of the controlled trial setting is important to evaluate how interventions are applied and assess the effectiveness of new treatments in routine clinical practice. Inclusion criteria are often rather restrictive compared with the patient populations seen by physicians in daily practice.

There are limited real-world data related to treatment with BV, and where it is available, efficacy and safety are consistent with those seen in clinical trials. Five retrospective observational studies have collected data for more than 200 patients with R/R HL treated with BV in centers in Asia, France, Italy, and Turkey.^12^-^16^ Across the studies, ORRs were in the range of 40-73%, and CRs were reported for 18-34% of patients. For the four studies that reported median progression-free survival (PFS), these ranged from 6.6 to 9.0 months. The most frequently observed adverse events across the studies included sensory neuropathy and neutropenia.

The present study investigates a population of patients who have received a median of 3 previous treatment regimens. These patients represent those who have relapsed and may then have a critical medical need, requiring a different management strategy to standard salvage therapy. We report the results of a retrospective, observational study with the objective of assessing the effectiveness and tolerability of BV for the treatment of R/R HL in a real-world setting, based on complementary data collected in a collaboration between institutes in the Czech Republic and Slovakia.

## Methods

This retrospective, observational study examined data for patients with R/R HL at eight centers in the Czech Republic and Slovakia. In the Czech Republic, data came from a clinical registry of patients with R/R HL who started treatment with BV between May 2013 and November 2015. An identical data collection strategy was used in Slovakia for patients who started BV treatment between July 2012 and January 2016. All patients were treated within government-funded schemes, with no patients included from clinical trials or named patient programs. Patients were CD30-positive, as determined by immunohistochemical examination on entry biopsy, typically with positivity in the membrane and Golgi apparatus in Hodgkin's and Reed-Sternberg cells of classical HL.

The analysis included data for patients who received BV for the treatment of R/R HL following ASCT or who were ineligible for ASCT after failure of one previous salvage therapy. BV was initiated at the recommended dosing of 1.8 mg/kg every 3 weeks for up to a maximum of 16 cycles.

The data collected and examined included patient characteristics and demographics, prior treatment history, details of BV treatment, clinical responses to BV treatment, and survival outcomes. Clinical responses were evaluated according to the revised Cheson criteria (2007),^17^ with all patients assessed by computed tomography before initiating BV and after 4, 8, 12, and 16 cycles of treatment as applicable. Use of positron emission tomography (PET) was not reimbursed within the government-funded schemes and was not routinely available. Overall survival (OS) was determined both from the time of HL diagnosis and the initiation of BV treatment.

Primary data were expressed using absolute and relative frequencies for categorical variables, and median and range (5-95 percentiles) for continuous variables. Differences between patient groups were assessed using the non-parametric Fisher exact test, Mann-Whitney U test, or Kruskal-Wallis test.

The analyses employed logistic regression models to quantify the association between potential predictors and principal, binary-coded endpoints (i.e. relapse rate, response to the therapy) using both univariate and multivariate-adjusted approaches. The models used maximum likelihood estimation, directly comparing the likelihood L0 for the null model where all slope parameters are zero, with the likelihood L1 of the fitted model. Significance of regression coefficients was tested using the Wald test statistic, which is based on the asymptotic normality property of maximum likelihood estimates (tested on the basis of Chi-square distribution). Similarly, univariate and multivariate Cox proportional hazard regression models were applied to test the impact of potential predictors on OS as a time-to-event endpoint. Both univariate and multivariate-adjusted estimates of odds ratio (OR) and hazard ratio (HR) were determined with 95% confidence intervals. Standard Kaplan-Meier curves were used to display OS profiles stratified according to clinical stage. Log-rank testing was used to assess the statistical significance of differences across the strata.

The study was conducted according to the principles expressed in the Declaration of Helsinki. Informed consent from the participants was not collected as all data were analyzed anonymously.

## Results

### Patient characteristics and treatment history

A total of 58 patients with R/R HL who had received treatment with BV were identified in the institutional databases and included in the retrospective analysis. Patient and disease characteristics, as well as the treatment history for the 58 patients, are summarized in **Table 1**. The median age of patients at diagnosis was 30.5 years, and the median follow-up from diagnosis was 4.3 years from diagnosis and 1.4 years from BV initiation.

Patients had received a median of 3 treatment regimens prior to treatment with BV. The majority of patients (86%) had previously received doxorubicin/bleomycin/vinblastine/dacarbazine (ABVD) at some point in the course of their treatment. Other frequently used chemotherapy regimens included bleomycin/etoposide/doxorubicin/cyclophosphamide/vincristine/procarbazine/prednisolone (BEACOPP), dexamethasone/high-dose cytarabine/cisplatin (DHAP) and ifosfamide/carboplatin/etoposide (ICE). The proportion of patients who had received radiotherapy was 69%. Most patients (91%) had received at least one ASCT and 12% had had an allogeneic stem cell transplant (allo-SCT). Of the patients receiving prior ASCT, 7 patients underwent 2 transplants prior to BV administration, three of which were tandem-ASCTs with an inter-transplant interval of 5-7 months. Based on the activity of the disease, patients in this study were divided into two groups prior to initiating BV - 52 patients (90%) were in relapse with progressive disease (PD) and 6 patients (10%) had partial stabilization (i.e. they had either partial response [PR] or stable disease [SD]). Patients with PR/SD receiving BV treatment were those whose prior therapy did not lead to CR, despite 5 of these 6 patients having undergone ASCT, two of which were tandem-ASCTs.

### Safety

All patients received at least 3 doses of BV with a median 7.5 cycles (range, 3-16 cycles). Only 1 patient (2%) required a reduction in the dose of BV to 1.2 mg/kg due to neutropenia (Grade 3/4), while dosing delays were instituted for 8 (14%) of the patients. Toxic effects of BV which lead to a prolongation of the dose interval included 2 cases of leukopenia (Grade 3/4), 1 case of urinary tract infection, 2 cases of acute bronchitis, 1 case of odontogenic infection and 1 case of increase in pancreatic amylase. In our retrospective study, the most common toxic manifestation of BV was neurological toxicity, which was observed in 21 patients (36.2%). Of these, 12 patients presented with peripheral sensory neuropathy (Grade 1/2) only, 3 patients with peripheral motor neuropathy (Grade 1/2) only, and 6 patients with simultaneous occurrence of sensory and motor neuropathy (Grade 1/2).

### Therapeutic responses to brentuximab vedotin

Patients received a median of 7.5 cycles of BV treatment. Of the 58 patients, 19 (33%) had a CR following BV treatment, with 8 (14%) having a PR and 31 (53%) having PD (**Table 2**). Discontinuation of BV was due to disease progression, negotiations with health insurance companies, or receipt of allo-SCT. Following treatment failure with BV, 6 patients received nivolumab, with 3 patients subsequently achieving CR. Of the remaining 3 patients, 2 progressed and 1 died of septic shock while on nivolumab therapy.

Of the 58 patients treated with BV, the duration of the therapeutic response was evaluated in 34 patients, with the median being 5 months. Of the remaining patients, 13 patients achieving a response to BV were directed to allo-SCT, and the remaining 11 patients are alive with no signs of relapse/progression of HL.

Patients who achieved a CR after BV subsequently received more cycles of treatment (median 8 cycles) as they did not relapse during BV treatment. Patients with an inadequate response to BV received fewer cycles (PR, median 5.5 cycles; PD, median 6 cycles) mostly due to the poor response (*P<*0.001). BV treatment was discontinued early (patients received <8 cycles of BV) in a total of 29 patients. Of these, 19 patients discontinued treatment due to insufficient response to BV. Six patients discontinued BV treatment early due to redirection to allo-SCT, of which 3 patients were in PR following 3, 5 and 6 cycles of BV, and 3 patients were in CR following 6, 3 and 6 cycles. The number of BV cycles was also influenced by negotiations with health insurance companies, which lead to the decision to discontinue BV treatment early in 2 patients who achieved CR. No patients discontinued treatment early due to toxicity or drug availability issues, and the remaining 2 patients discontinued treatment early due to unknown reasons.

Patients receiving fewer previous treatment regimens were more likely to achieve a CR. Univariate and multivariate logistic regression models confirmed these associations, and that none of the other factors tested were significantly correlated with the likelihood of achieving a CR (data in **Supplementary Table S1**).

No factor was found to be significantly associated with the risk of relapse in univariate or multivariate logistic regression models (**Supplementary Table S2**). Overall, 14 patients underwent allo-SCT after BV treatment. CR was achieved following allo-SCT in all patients who received the transplant in CR (6 patients) or PR (3 patients). Of the remaining 5 patients receiving allo-SCT with PD, CR was achieved in 2 cases.

### Progression-free survival

**Figure 1:**
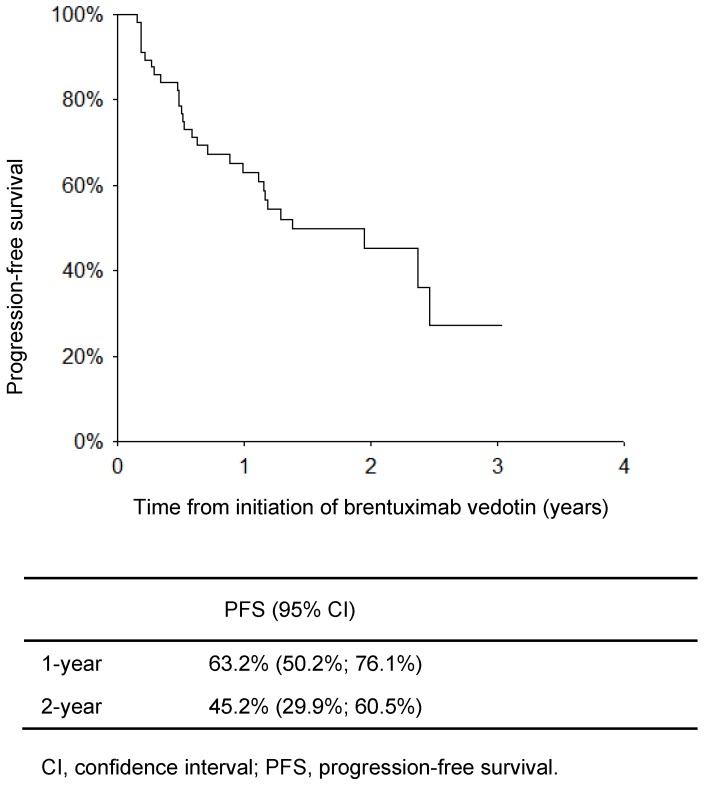
Progression-free survival of patients with Hodgkin lymphoma from initiation of brentuximab vedotin

**Figure 1** shows the PFS for the 58 patients from the start of BV therapy. The PFS rates at 1 and 2 years were 63.2% and 45.2%, respectively. The median PFS was 1.38 (0.56-2.21) years.

### Overall survival

**Figure 2:**
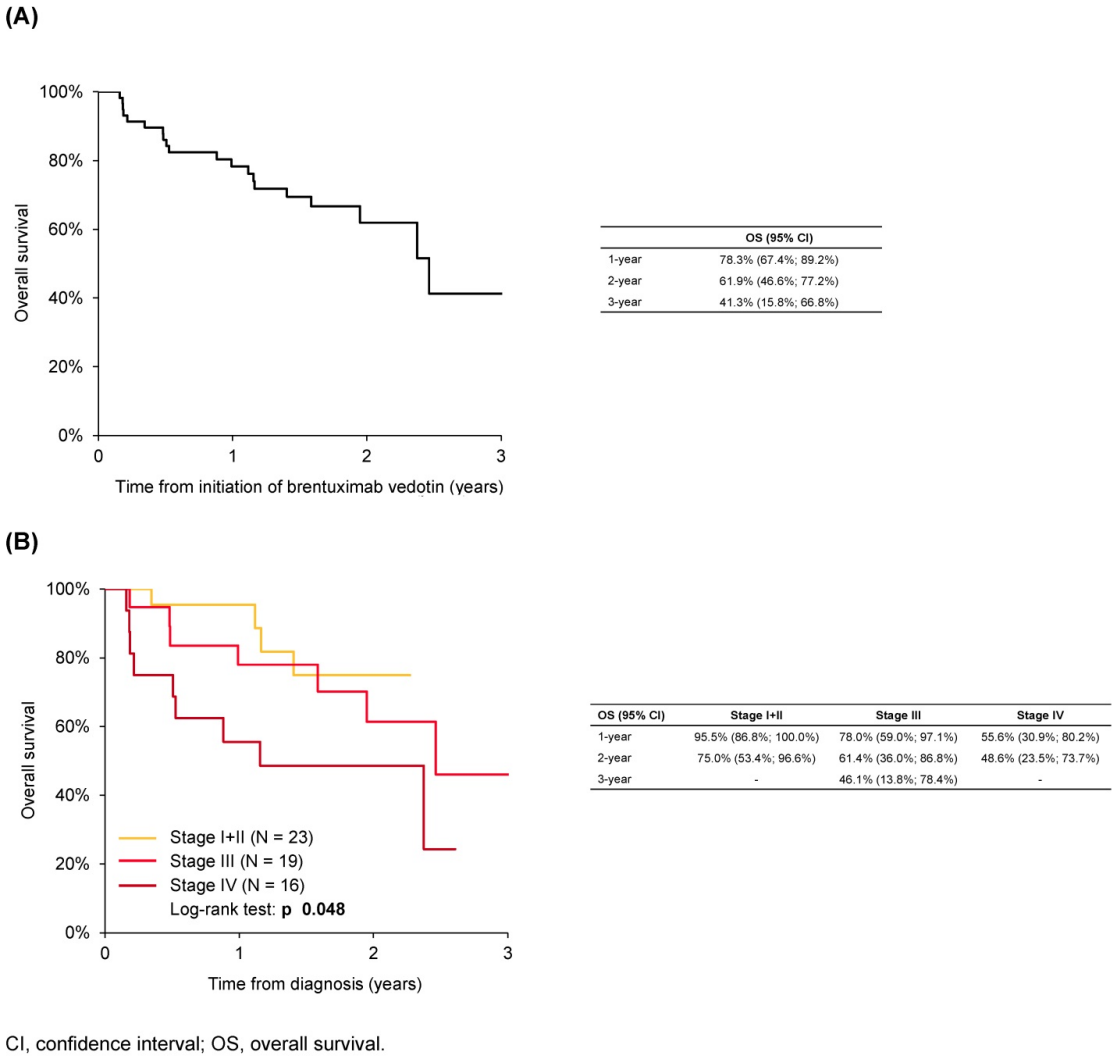
(A) Overall survival of patients with Hodgkin lymphoma from initiation of brentuximab vedotin and (B) according to disease stage

At 1 and 3 years after initiation of BV, OS was 78% and 41%, respectively (**Figure 2A**). The OS rate at 1 year after BV initiation was 96% for patients with Stage I or II disease at diagnosis, compared with 78% for Stage III disease and 56% for Stage IV disease (**Figure 2B**). In a univariate analysis, OS from initiation of BV was significantly shorter in patients initially diagnosed with more advanced stage HL, and significantly longer in patients who received six or more cycles of BV compared with those who received five or fewer cycles (*P*<0.05; **Supplementary Table S3**).

## Discussion

Our study reports and assesses data related to the treatment of R/R HL, and specifically BV, in clinical practice in a 'real-world' setting. This information can provide a valuable complement to findings from randomized clinical trials, capturing experience in routine clinical practice rather than the selected, highly controlled trial conditions.

The characteristics of the patients included in our study were typical of those encountered in real-life practice who are eligible for treatment with BV. Comparing our observational study with the pivotal phase II clinical trial of BV in R/R HL, the median age of patients (30.5 and 31 years, respectively) was the same, and the median number of prior chemotherapy regimens (3.0 and 3.5, respectively), proportion of patients who had previously received at least one ASCT (both 91%) and the proportion who had received radiotherapy (69% and 67%, respectively) were all similar.^8^ Some differences exist between the study populations: in the pivotal phase II trial, for example, all patients had undergone ASCT, whereas the present study included 5 patients who were ineligible for ASCT. Response assessment also differed: while the phase II trial included both computed tomography and PET scans in the trial design, the present study evaluated response by computed tomography only - PET scans were not routinely available in this retrospective analysis. While results may have been different with a PET-driven approach, this study was not a clinical trial, and PET/CT is not always a standard procedure for interim disease response evaluation in routine clinical practice, even in countries other than the Czech Republic and Slovakia.

Most patients (86%) had received an ABVD regimen, and a substantial proportion had received BEACOPP (36%), as part of their treatment history prior to BV, reflecting that some patients were switched from BEACOPP to ABVD due to toxicity issues. This suggests that practice in the centers in the Czech Republic and Slovakia is consistent with ESMO guidelines, which recommend either ABVD or BEACOPP as front-line treatment for newly diagnosed HL.^3^ Frequently used salvage regimens included DHAP and ICE. The high proportion of patients who had previously received at least one ASCT (91%), with the exceptions being those unsuitable for ASCT, reflects that use of BV followed the licensed indications for BV as well as ESMO recommendations.^3^,^18^

Seven patients (12%) had received prior allo-SCT, consistent with ESMO guidelines that this is not a standard approach in HL but can be considered for young patients in good general condition with relapse after ASCT.^3^ Of these 7 patients, 4 achieved CR following subsequent treatment with BV, none of whom relapsed. BV treatment in patients post-allo-SCT has thus far not been studied extensively. A previous study of 25 patients with HL relapsing after allo-SCT, indicated that BV treatment had potential utility in post-allo-SCT management, resulting in a 38% CR rate.^20^ Although a small number, the results of the present study provide further support for the efficacy of BV treatment post-allo-SCT.

The response rates after treatment with BV are mostly consistent with previous studies. For example, the CR rate of 33% following BV treatment among patients in our clinics in the Czech Republic and Slovakia was the same as that seen in a long-term follow-up of patients in the pivotal phase II trial.^9^ The ORR (47%) and CR rates in our study were also within the range reported from other observational studies (ORR: 40-73%; CR: 18-34%).^12^-^16^ Based on the analysis of experience in our clinics, patients who received less extensive prior treatment were more likely to achieve CR, and subsequently a higher number of cycles of BV treatment. There was some indication that the risk of relapse was increased when the dosing interval of BV was changed from that recommended, but the small number of patients for whom relevant data were available limits interpretation. In patients with PD after BV, there was a longer interval between BV cycles. The cause of prolongation between BV cycles was most often due to infectious complications (respiratory tract infections) and myelotoxicity. It is not currently clear how to distinguish between those patients who are likely to gain the greatest benefit from BV treatment (i.e. long-term remission) and therefore do not require any further consolidation and those for whom BV therapy should serve as a bridge to allo-SCT. A recent review of published reports from the named patient program for BV, drawing together experience from 480 patients with R/R HL in approximately 60 countries, found 1-year OS rates of 67-76% and 2-year OS rates of 58-67% from the initiation of BV treatment.^21^ Survival rates in our study were consistent with the results from the named patient program, with 1- and 2-year OS rates of 78% and 62%, respectively. Comparing these results in real-world settings with those from clinical trials, in the pivotal phase II trial with BV, the estimated 1-year OS rate was somewhat higher at 89% and in long-term follow-up the 5-year OS rate was 41%.^8^,^9^ The duration of therapeutic response (5 months) appears to be lower than that observed in previous pivotal and real-world studies.^8^,^12^,^13^,^15^,^21^ This may be due to the inclusion of more heavily pretreated patients with R/R HL, who had previously undergone 2-9 previous chemotherapy regimens, as well as prior ASCT and allo-SCT.

By drawing on real-world data, our study provides further evidence on the use and effectiveness of BV in routine clinical practice. The use of BV in R/R HL management should be considered in the context of other treatment options, such as immune checkpoint inhibitors.^22^-^25^ Data were collected using a consistent, standardized and systematic approach that allowed a robust analysis. At the same time, common to other real-world analyses, our study is potentially open to numerous confounding factors and sources of bias that would, to some extent, be reduced by the restrictions and controls of a prospective clinical trial. The number of patients included in the analysis was also relatively small, reducing the scope for sub-analyses and interpretation, which means that valid conclusions can only be drawn from a limited number of statistical tests. In conclusion, this analysis of real-world data for patients with R/R HL in the Czech Republic and Slovakia, demonstrated efficacy with BV consistent with that seen in previous reports from real-world practice as well as clinical trials.

## Supplementary Material

Supplementary tables.

## Figures and Tables

**Table 1:** Patient characteristics and treatment history

		N (%)	Median (5-95 percentiles)
**Patient characteristics**			
Length of follow-up (years)		4.3	(1.2-16.0)
Age at time of diagnosis		30.5	(20.0-53.0)
Sex	Men	33	(57)
	Women	25	(43)
HL stage	I+II	23	(40)
	III	19	(33)
	IV	16	(28)
Indication for BV	Relapse after autologous transplantation	53	(91)
	R/R, unsuitable for ASCT	5	(9)
**Pretreatment**			
Number of regimens		3.0	(2.0-9.0)
Previous regimens^a^	ABVD	50	(86)
	BEACOPP	21	(36)
	COPP	15	(26)
	DHAP	26	(45)
	Gemcitabine	9	(16)
	ICE	26	(45)
	IGEV	9	(16)
	Other	29	(50)
Radiotherapy	No	18	(31)
	Yes	40	(69)
ASCT	No	5	(9)
	Yes - 1 transplant	46	(79)
	Yes - 2 transplants	7	(12)
Allo-SCT	No	51	(88)
	Yes	7	(12)
Response before BV	PD/relapse	52	(90)
	PR/SD	6	(10)

**^a^**Treated with regimen at any stage in treatment history; only regimens assigned to at least five patients are listed. ABVD: doxorubicin, bleomycin, vinblastine and dacarbazine; allo-SCT: allogenic stem cell transplantation; ASCT: autologous stem cell transplantation; BEACOPP: bleomycin, etoposide, doxorubicin, cyclophosphamide, vincristine, procarbazine and prednisolone; BV: brentuximab vedotin; COPP: cyclophosphamide, vincristine, procarbazine and prednisolone; DHAP: dexamethasone, high-dose cytarabine and cisplatin; HL: Hodgkin lymphoma; ICE: ifosfamide, carboplatin and etoposide; IGEV: ifosfamide, gemcitabine and vinorelbine; PD: progressive disease; PR: partial response; R/R: relapsed/refractory; SD, stable disease.

**Table 2:** Treatment and outcomes after brentuximab vedotin treatment

		N (%)	Median (5-95 percentiles)
**BV treatment**			
Number of BV cycles		7.5	(3.0-16.0)
Number of BV cycles	2-5	20	(35)
	6-10	29	(50)
	11-16	9	(16)
Dose reduction	No	57	(98)
	Yes	1	(2)
Interval change^a^	No	16	(67)
	Yes	8	(33)
Response during treatment	CR	8	(14)
	PR	26	(45)
	SD	7	(12)
	PD	12	(21)
	Not available	5	(9)
**Outcome**			
Response on BV	CR	19	(33)
	PR	8	(14)
	PD	31	(53)
Relapse^b^	No	21	(78)
	Yes	6	(22)
Death	No	38	(66)
	Yes	20	(35)

Data are presented as N (%) or median (5-95 percentiles). ^a^Data are not available for 34 patients. ^b^Relapse occurrence was assessed only in patients with a CR or PR on BV treatment. BV: brentuximab vedotin; CR: complete response; PD: progressive disease; PR: partial response; SD: stable disease.
