# Supplementary material for: Treatment of Relapsed/Refractory Hodgkin Lymphoma: Real-World Data from the Czech Republic and Slovakia
Source: J Cancer. 2019 Aug 28;10(21):5041–8. doi: 10.7150/jca.29308 (PMC6775599; doi:10.7150/jca.29308)
Supplement: Supplementary file 1 — Supplementary tables. [file jcav10p5041s1.pdf]

Supplementary Appendix

Table S1: Therapeutic responders with brentuximab vedotin: univariate and multivariate logistic regression models

Predictors		Univariate models		Multivariate adjusted model[a]	
		OR (95% CI)	*P*	OR (95% CI)	*P*
Sex	Men	Ref.		Ref.	
	Women	1.778 (0.587–5.382)	0.309	1.937 (0.418–8.976)	0.398
Age at time of diagnosis	<25	Ref.		Ref.	
	≥25	0.960 (0.307–2.998)	0.944	0.951 (0.201–4.494)	0.949
HL stage	I+II	Ref.		Ref.	
	III	0.907 (0.259–3.177)	0.879	0.504 (0.079–3.208)	0.468
	IV	0.359 (0.079–1.623)	0.183	0.178 (0.019–1.632)	0.127
Indication for BV	Relapse after ASCT	Ref.		Ref.	
	R/R, unsuitable for ASCT	0.486 (0.051–4.676)	0.532	-	-
Number of previous regimens	2	Ref.		Ref.	
	>2	0.151 (0.045–0.507)	**0.002**	0.212 (0.049–0.911)	**0.037**
Previous ASCT	No	Ref.		Ref.	
	Yes – 1 transplant	1.750 (0.179–17.101)	0.630	3.894 (0.060–253.325)	0.523
	Yes – 2 transplants	5.333 (0.375–75.776)	0.216	10.766 (0.138–839.536)	0.285
Previous allo-SCT	No	Ref.		Ref.	
	Yes	3.200 (0.637–16.066)	0.158	7.227 (0.710–73.592)	0.095
Number of BV cycles	2–5	Ref.		Ref.	
	6–10	3.463 (0.822–14.593)	0.091	4.599 (0.718–29.462)	0.107
	11–16	7.083 (1.172–42.793)	**0.033**	15.172 (1.157–198.967)	**0.038**
Dose reduction	No	Ref.		Ref.	
	Yes	-	-	-	-
Interval change[b]	No	Ref.		Ref.	
	Yes	0.556 (0.084–3.690)	0.543	-	-

[a]Calculation of multivariate adjusted model is based on predictors which are not redundant and do not contain missing values. [b]Data are not available for 34 patients.

allo-SCT: allogenic stem cell transplantation; ASCT: autologous stem cell transplantation; BV: brentuximab vedotin; CI: confidence interval; HL: Hodgkin lymphoma; OR: odds ratio; PD; progressive disease; PR: partial response; Ref.: reference value; R/R: relapsed/refractory; SD: stable disease.

Table S2: Relapse occurrence after brentuximab vedotin in univariate and multivariate logistic regression models

Predictors		Univariate models		Multivariate adjusted model[a]	
		OR (95% CI)	*P*	OR (95% CI)	*P*
Sex	Men	Ref.		Ref.	
	Women	2.667 (0.397–17.914)	0.313	3.510 (0.328–37.594)	0.299
Age at time of diagnosis	<25	Ref.		Ref.	
	≥25	0.308 (0.045–2.083)	0.227	0.118 (0.008–1.654)	0.113
HL stage	I+II	Ref.		Ref.	
	III+IV	1.500 (0.223–10.077)	0.677	3.503 (0.193–63.630)	0.397
Indication for BV	Relapse after ASCT	Ref.		Ref.	
	R/R, unsuitable for ASCT	-	-	-	-
Number of previous regimens	2	Ref.		Ref.	
	>2	1.333 (0.216–8.219)	0.757	1.964 (0.200–19.259)	0.562
Previous ASCT	No	Ref.		Ref.	
	Yes – 1 transplant	-	-	-	-
	Yes – 2 transplants	-	-	-	-
Previous allo-SCT	No	Ref.		Ref.	
	Yes	-	-	-	-
Number of BV cycles	2–5	Ref.		Ref.	
	6–10	0.417 (0.045–3.838)	0.440	0.317 (0.016–6.265)	0.450
	11–16	1.250 (0.118–13.240)	0.853	0.741 (0.032–17.172)	0.852
Dose reduction	No	Ref.		Ref.	
	Yes	-	-	-	-
Interval change[b]	No	Ref.		Ref.	
	Yes	-	-	-	-

[a]Calculation of multivariate adjusted model is based on predictors which are not redundant and do not contain missing values. [b]Data are not available for 34 patients.

allo-SCT: allogenic stem cell transplantation; ASCT: autologous stem cell transplantation; BV: brentuximab vedotin; CI: confidence interval; HL: Hodgkin lymphoma; OR: odds ratio; PD; progressive disease; PR: partial response; Ref.: reference value; R/R: relapsed/refractory; SD: stable disease.

Table S3: Overall survival from diagnosis and initiation of brentuximab vedotin treatment: univariate Cox regression models (N = 58)

**Predictors**		**Time from diagnosis**		**Time from initiation of BV**	
		**HR (95% CI)**	***P***	**HR (95% CI)**	***P***
Sex	Men	Ref.			
	Women	0.531 (0.191–1.479)	0.226	0.457 (0.175–1.194)	0.110
Age at time of diagnosis	<25	Ref.		Ref.	
	≥25	1.615 (0.618–4.216)	0.328	1.407 (0.537–3.691)	0.487
HL stage	I+II	Ref.		Ref.	
	III	2.379 (0.692–8.180)	0.169	1.561 (0.442–5.506)	0.489
	IV	3.788 (1.160–12.366)	**0.027**	3.274 (1.013–10.807)	**0.048**
Indication for BV	Relapse after ASCT	Ref.		Ref.	
	R/R, unsuitable for ASCT	1.712 (0.390–7.519)	0.477	1.726 (0.396–7.523)	0.468
Number of previous regimens	2	Ref.		Ref.	
	>2	2.273 (0.658–7.853)	0.194	3.121 (0.913–10.669)	0.070
Previous ASCT	No	Ref.		Ref.	
	Yes – 1 transplant	0.683 (0.154–3.028)	0.616	0.644 (0.147–2.820)	0.560
	Yes – 2 transplants	0.162 (0.014–1.858)	0.144	0.208 (0.019–2.318)	0.202
Previous allo-SCT	No	Ref.		Ref.	
	Yes	1.369 (0.398–4.704)	0.618	1.017 (0.289–3.577)	0.979
Number of BV cycles	2–5	Ref.		Ref.	
	6–10	0.446 (0.172–1.161)	0.098	0.330 (0.121–0.899)	**0.030**
	11–16	0.230 (0.049–1.079)	0.062	0.200 (0.041–0.970)	**0.046**
Dose reduction	No	Ref.		Ref.	
	Yes	-	-	-	-
Interval change[a]	No	Ref.		Ref.	
	Yes	0.861 (0.187–3.959)	0.847	1.338 (0.297–6.021)	0.704

[a]Data are not available for 34 patients.

allo-SCT: allogenic stem cell transplantation; ASCT: autologous stem cell transplantation; BV: brentuximab vedotin; CI: confidence interval; HL: Hodgkin lymphoma; HR: hazard ratio; PD; progressive disease; PR: partial response; Ref.: reference value; R/R: relapsed/refractory; SD: stable disease.
